# Nanoparticles Formulations of Artemisinin and Derivatives as Potential Therapeutics for the Treatment of Cancer, Leishmaniasis and Malaria

**DOI:** 10.3390/pharmaceutics12080748

**Published:** 2020-08-09

**Authors:** Sibusiso Alven, Blessing Atim Aderibigbe

**Affiliations:** Department of Chemistry, University of Fort Hare, Alice Eastern Cape 5700, South Africa; 201214199@ufh.ac.za

**Keywords:** cancer, malaria, leishmaniasis, arteminisin, nanoparticles, nanocarriers

## Abstract

Cancer, malaria, and leishmaniasis remain the deadly diseases around the world although several strategies of treatment have been developed. However, most of the drugs used to treat the aforementioned diseases suffer from several pharmacological limitations such as poor pharmacokinetics, toxicity, drug resistance, poor bioavailability and water solubility. Artemisinin and its derivatives are antimalarial drugs. However, they also exhibit anticancer and antileishmanial activity. They have been evaluated as potential anticancer and antileishmanial drugs but their use is also limited by their poor water solubility and poor bioavailability. To overcome the aforementioned limitations associated with artemisinin and its derivatives used for the treatment of these diseases, they have been incorporated into nanoparticles. Several researchers incorporated this class of drugs into nanoparticles resulting in enhanced therapeutic outcomes. Their potential efficacy for the treatment of parasitic infections such as malaria and leishmaniasis and chronic diseases such as cancer has been reported. This review article will be focused on the nanoparticles formulations of artemisinin and derivatives for the treatment of cancer, malaria, and leishmaniasis and the biological outcomes (in vitro and in vivo).

## 1. Introduction

The mortality rate of cancer, malaria, leishmaniasis remains high around the world although various approaches have been developed for their treatment. Cancer is a chronic disease that is recognized by an abnormal uncontrolled growth of any type of human body cells [1,2]. The causes of cancer are classified into two groups: external factors (such as pollution, smoking, radiation, and infectious pathogens etc.) and internal factors (such as hormones, immune conditions, and genetic mutations) [2]. Among the various types of cancers, breast, lung, and colorectal cancer are the common types of cancers [3]. In 2018, it was estimated that there were 18.1 million cases of cancer with 9 million deaths worldwide [4]. The strategies that have been employed for the treatment of cancer include chemotherapy (anticancer drugs), surgery, immunotherapy, hormonal therapy, radiotherapy. However, the stage and the environment of the tumor determine the treatment approach [5,6]. The use of chemotherapeutic drugs is the most common approach used for the treatment of cancer [6].

On the other hand, for parasitic infectious diseases such as malaria and leishmaniasis, treatment is very challenging. Malaria is a vector and blood-borne infectious disease that is caused by plasmodium parasite inoculation from a female infected anopheles mosquito. *P. falciparum*, the most lethal malaria pathogen causes life-threatening symptoms followed by *P. vivax* [7]. The severe symptoms of *P. falciparum* malaria include cerebral malaria, adult respiratory distress syndrome (ARDS), coma, seizures, and disseminated intravascular coagulation [8]. The people that are at a high risk of malaria infection are pregnant women and children under the age of 5 years due to changes in their immune system. World Health Organization (WHO) reported 217 million malaria cases in 2016 which increased to 219 million cases with 435,000 deaths in 2017 [9]. The use of antimalarial drugs is an effective approach for the treatment of malaria. However, their use is restricted by drug toxicity, drug resistance, poor bioavailability, and pharmacokinetics. Antimalarial drugs can be classified based on their chemical structures such as artemisinin derivatives, 4-aminoquinolines, hydroxynaphthoquinones, diaminopyrimidines, 4-quinolinemethanols, sulfonamides, 8-aminoquinolines, and quinolines-based cinchona alkaloids [10].

Leishmaniasis is an infectious vector-borne disease caused by a protozoan parasite called *Leishmania* which is transmitted through an infected sand fly and there are approximately 21 species of *Leishmania* that infect people [11,12]. This infectious disease is endemic in the tropical and subtropical regions including southern Europe, both Americas, Africa, and Asia. It can be transmitted from infected individuals via needle sharing, blood transfusion, and from mother to baby during gestation although these cases are very rare [11]. World Health Organization (WHO) reported that leishmaniasis is among the most neglected tropical diseases, with an estimated 700,000 to 1 million new cases annually. Over 12 million people are infected with the disease in 98 countries and 2 million new cases are predicted to occur every year [13,14]. Leishmaniasis is classified into three clinical forms in humans namely visceral, mucocutaneous, and cutaneous. Visceral leishmaniasis which is also known as kala-azar is the most chronic form of the disease [15]. There are currently no safe and effective drugs available for the treatment of leishmaniasis. Most of the presently used antileishmanial drugs suffer from several limitations such as toxicity, drug resistance, and hospitalization requirement [16].

Malaria and leishmaniasis are common amongst the low and middle-income countries of Africa, Asian and Latin America [17,18]. In these areas, people do have access to clean water and there is lack of proper disposal of wastes. The people are also exposed to unsanitary environments which are breeding grounds for the parasites [18]. Cancer, on the other hand, has been reported to be the second leading cause of deaths by WHO. Over 70% of deaths from cancer have been reported in the low- and middle-income countries resulting from inaccessibility to early diagnosis and treatment. The economic impact of cancer, malaria and leishmaniasis is increasing and high [18,19]. Due to the high cases of malaria, leishmaniasis and cancer-related deaths in the low and middle-income countries, there is a pressing need to developed effective therapeutics which are affordable and accessible. There are currently internationally approved treatment plans for these diseases.

Artemisinin is effective against cancer and some parasitic diseases and it is a sesquiterpene with a unique peroxide bridge which plays an important role in its active biological mode of action. It suffers from poor bioavailability and its derivatives such as artesunate, artemether, dihydroartemisinin etc. are recommended for clinical use. Furthermore, artemisinin and its derivatives pharmacological properties can be improved by incorporating them into nanocarriers. Some nanocarriers include nanoparticles [20], dendrimers [21], nanomicelles [22], polymer-drug conjugates [23], hydrogels [24], nanogels [25], nanocapsules [26], and nanoliposomes [27], have been designed for the incorporation of artemisinin and its derivatives. These nanocarriers are very useful in the field of drug delivery [28]. The advantages of nanocarriers in the field of oncology and parasitic infections (such as malaria and leishmaniasis) include reduced/non-drug toxicity, the overcoming of drug resistance, improved aqueous drug solubility, enhanced bioavailability and pharmacokinetics, specific-site drug delivery, drug cellular internalization etc. [29]. There are several reports and evaluations of nanoparticles for drug delivery of artemisinin and its derivatives against cancer, malaria and leishmaniasis. The results are promising and suggest that nanoparticles loaded with artemisinin and its derivatives are potential therapeutics for the treatment of chronic and parasitic diseases. Due to the positive therapeutic reports from several researchers on nanoparticles loaded with artemisinin and derivatives, this review gives a comprehensive report of nanoparticles loaded with artemisinin and derivatives. This review is focused only on three types of nanoparticles: polymeric, metal-based, and lipid nanoparticles.

## 2. Anticancer Drugs

### 2.1. Classification of Anticancer Drugs

**Figure 1:**
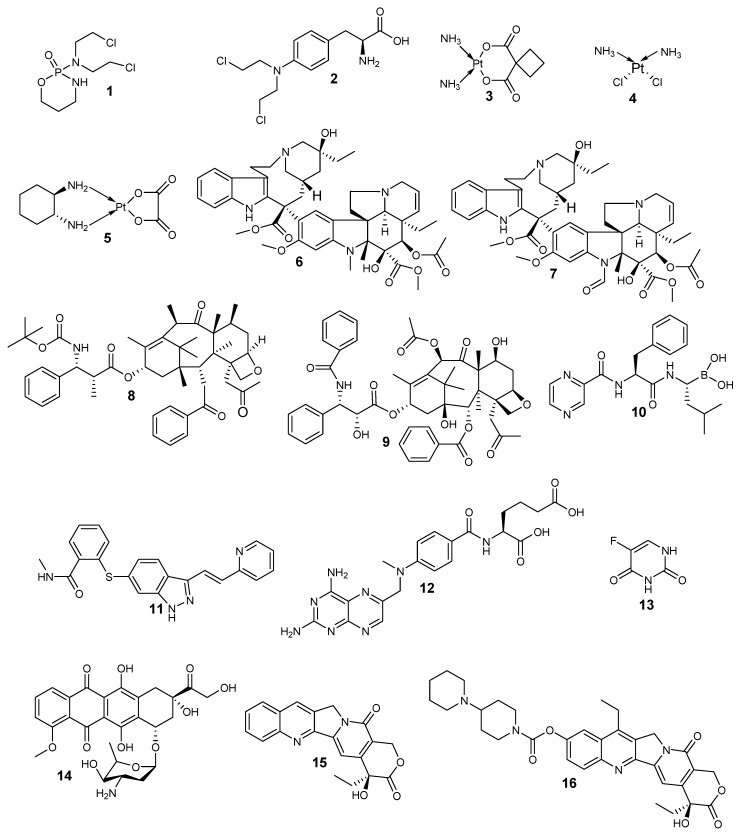
Structures of the four classes of chemotherapeutic agents (cyclophosphamide **1**, melphalan **2**, carboplatin **3**, cisplatin **4**, oxaliplatin **5**, vinblastine **6**, vincristine **7**, docetaxel **8**, paclitaxel **9**, bortezomib **10**, bevacizumab **11**, methotrexate **12**, fluorouracil **13**, doxorubicin **14**, camptothecin **15**, and irinotecan **16**).

Chemotherapeutic agents are classified into four groups based on their mode of action: alkylating agents, anti-tubulin agents, antimetabolites, and topoisomerase inhibitors (Figure 1). Alkylating agents bind covalently with the Deoxyribonucleic acid (DNA) and crosslink them resulting in strand breaks of DNA causing base pairing that is abnormal thereby hindering cell division and cell death. They are effective in all phases of the cell cycle and are used to treat different types of cancers [30,31]. Examples of alkylating agents include cyclophosphamide **1**, melphalan **2**, carboplatin **3**, cisplatin **4**, and oxaliplatin **5**. The second class of anticancer drugs are anti-tubulin agents which are responsible for the disruption of mitotic spindles and terminate cell division by a process known as mitosis [32]. Examples of anti-tubulin agents are vinblastine **6**, vincristine **7**, docetaxel **8**, and paclitaxel **9**. The antimetabolites inhibit nucleic acid biosynthesis, and some examples include bortezomib **10**, bevacizumab **11**, methotrexate **12**, and fluorouracil **13** [33,34]. Topoisomerase inhibitors inhibit DNA replication [35]. These inhibitors act by binding to the topoisomerase active sites thereby hindering the binding of the DNA substrate [36,37]. Examples of topoisomerase inhibitors include doxorubicin **14**, camptothecin **15**, and irinotecan **16**.

**Figure 2:**
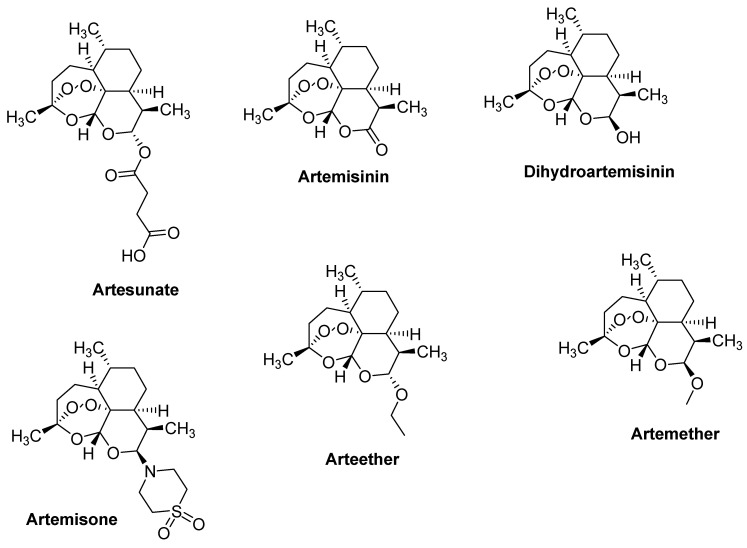
Some structures of artemisinin and derivatives with anticancer activity.

Artemisinin and its derivatives exhibit anticancer activity (Figure 2). The iron-mediated cleavage of the endoperoxide bridge of artemisinin and its derivatives plays a crucial role in their anti-cancer properties. It has been reported that cancer cells have more intracellular free iron when compared to healthy/normal cells. Artemisinin endoperoxide moiety reacts with the intracellular free iron to form cytotoxic radicals [38]. Furthermore, artemisinin and its derivatives have been reported to cause apoptosis in many cancer cell lines [39,40,41]. Their mode of action as anticancer agents also includes the alteration of oxidative damage reactions, apoptotic response, by suppressing cells proliferation, by the inhibition of cell invasion and metastasis, arresting the tumor cell cycle, by disrupting the cancer signaling pathway, hindering angiogenesis, and by regulating the tumor microenvironment [42,43,44,45,46,47]. The combination of two or more of the aforementioned mechanisms have been reported to contribute to the anticancer activity of artemisinin and its derivatives as shown in Table 1.

### 2.2. Multi-Drug Resistance of Anticancer Drugs

Drug resistance of anticancer drugs limits their ability to inhibit tumor growth. The principle of mechanisms may involve changed membrane transport including the product of p-glycoprotein of the multidrug resistance gene as well as other related proteins, changed target enzyme, decreased activation of the drug, increased degradation of the drug because of changed manifestation of drug breaking enzymes, drug interaction, subcellular redistribution, improved repair of DNA, and apoptosis failure due to mutation in cell cycle proteins [69,70]. Combination therapy is one of the strategies that is used to overcome chemotherapy resistance by utilizing different types of drugs with low toxicities for maximal dosage. Gene knockout employing antisense molecules has been reported to hinder drug resistance [69].

## 3. Antimalarial Drugs

### 3.1. Classification of Antimalarials Based on Their Mode of Action in the Malaria Life Cycle

**Figure 3:**
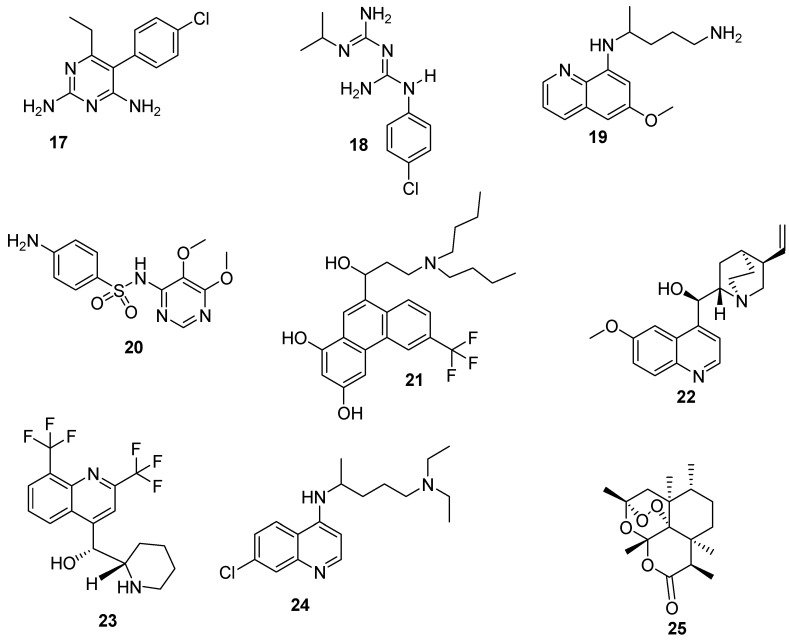
Antimalarial drugs used to target the four stages of the malaria parasite (pyrimethamine **17**, proguanil **18**, primaquine **19**, sulfadoxine **20**, halofantrine **21**, quinine **22** and mefloquine **23**, chloroquine **24** and artemisinin **25**).

The drugs of choice for the treatment of malaria depend on the four stages of the plasmodium life cycle in the vector (female anopheles mosquito) and the host (human). The four stages of the malaria life cycle are liver, blood, transmission, and mosquito stage. The antimalarial drugs that can be used in each stage are shown in Figure 3.

1. Liver stage: The female mosquito injects the plasmodium parasites in the form of sporozoites into the human bloodstream. The sporozoites invade the liver and multiply to form schizonts. *P. ovale* and *P. vivax* species that can stay dormant in the liver in the form of hypnozoites resulting in relapse after months or years of initial infection. The antimalarial drugs that can be used to target this stage must be able to prevent the development of malaria and those drugs are called prophylactic treatment e.g., pyrimethamine **17**, proguanil **18** etc. In addition, tissue schizonticides are also needed at this stage for a radical cure and they are active against hypnozoites. Good examples of a tissue schizonticides are primaquine **19**, artemisinin **25** etc. [71].

2. Blood stage: After some days, approximately 5–10 days, there is a formation of merozoites that invade the erythrocytes resulting in the symptomatic terrible fevers. The merozoites in their intraerythrocytic phase undergo different forms (trophozoites, rings, and schizonts) to produce a number of merozoites that are released into the blood to infect new erythrocytes. The antimalarial drugs that can be used to target this stage terminate the symptoms of malaria and related mortality. The antimalarial drugs used are known as blood schizontocides e.g., sulfadoxine **20**, halofantrine **21**, quinine **22** and mefloquine **23** [10,72].

3. Transmission stage: During the replication of merozoites, some of them differentiate in the bone marrow to form sexual (female and male) gametocytes. The female and male gametocytes are ingested by plasmodium-free female anopheles mosquito during a blood meal. The gametocytocidal drugs used to target this stage destroys the growth and development of sexual gametocytes such as chloroquine **24** and artemisinin **25** [73].

4. Mosquito stage: In this stage, the female and male gametocytes fuse together in the gut of the female anopheles mosquito to produce a zygote that further matures into an oocyst. The oocytes undergo several divisions to form sporozoites that are ready for the next host. The antimalarials that are used to target this stage are called schizonticides e.g., primaquine **19** and pyrimethamine **17**. They act by preventing the transfer of the disease to a human host [74,75].

Malaria is treated effectively by combination therapy. WHO approved the combination of antimalarial drugs for the treatment of uncomplicated *P. falciparum* which are artemether-lumefantrine; artesunate-amodiaquine; artesunate-mefloquine; dihydroartemisinin-piperaquine and artesunate-sulfadoxine-pyrimethamine [76,77]. Artemisinin derivatives clear the parasites rapidly from the blood at a factor of 10,000 in a 48 h asexual cycle. It is active against the sexual stage of the parasites thereby overcoming further transmission to the mosquitoes. The partner drugs that exhibit a longer elimination half-life are useful in clearing the remaining parasites thereby preventing the parasites from developing resistance to artemisinin. The partner drugs also act by providing a period of post-treatment prophylaxis [77,78].

### 3.2. Drug Resistance of Antimalarial Drugs

Most antimalarial drugs are active against the plasmodium parasite but almost all of them suffer from drug resistance. The common *Plasmodium* species that result in drug resistance is *P. falciparum*. It causes life-threatening symptoms such as cerebral malaria, anemia, severe fever, adult respiratory distress syndrome, seizure, disseminated intravascular coagulation, and coma [28]. The antimalarial drugs suffer from drug resistance when *Plasmodium* parasites survive and grow in the presence of administered drugs that are usually used to destroy and inhibit their growth. The following factors influence the drug resistance which is common with most antimalarial drugs: pharmacokinetic mismatch of antimalarial drugs, poor patient compliance and incorrect dosage [28].

The mechanism of drug resistance of the clinically used antimalarial drug such as chloroquine, is related to a high rate of drug efflux whereby the chloroquine-resistant plasmodium parasite release the pre-accumulated chloroquine 50 times faster when compared to the chloroquine-sensitive plasmodium parasites [79]. Another study revealed that the resistance of malarial parasites to chloroquine is caused by a reduced affinity of chloroquine to heme to form a heme-chloroquine complex [80]. Primaquine suffers from drug resistance because of trophozoites in the blood-stage that have mitochondrial-equivalent organelles which can reduce the antimalarial activity of primaquine [81].

The drug resistance of other antimalarial drugs results from gene mutations in *Plasmodium* parasite. For example, the genes that result in drug resistance in quinine, mefloquine, and amodiaquine are pfmdr1 (*P. falciparum* multidrug resistance transporter 1), pfnhe1 (*P. falciparum* proton/sodium exchanger 1), and pfcrt (*P. falciparum* chloroquine resistance transporter) [82]. Furthermore, drug resistance in antifolates, sulfadoxine, and pyrimethamine, is due to a point mutation on gene encoding for the dihydropteroate synthase (DHPS) and dihydrofolate reductase (DHFR), respectively [83]. The drug resistance of artemisinin drugs is multigenic with some resemblance with the quinolines as reported in vitro in a sequence of *Plasmodium* parasite isolate [84].

## 4. Antileishmanial Drugs

### 4.1. Classification of Antileishmanial Drugs

**Figure 4:**
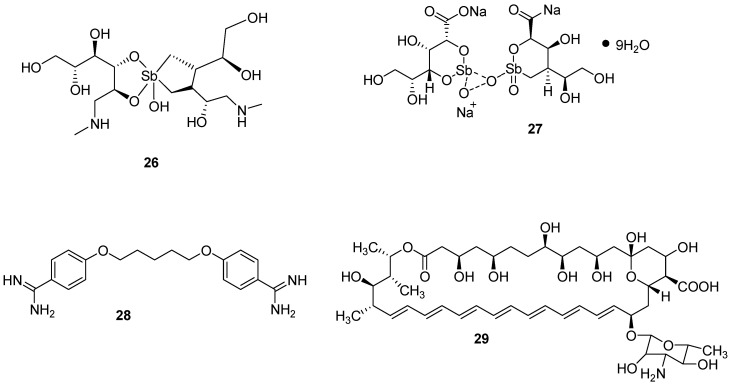
Drugs used for the treatment of leishmaniasis (meglumine antimoniate **26**, sodium stibogluconate **27**, pentamidine **28**, and amphotericin B **29**).

The currently used drugs for the treatment of leishmaniasis are classified into three groups: pentavalent antimonials (e.g., meglumine antimoniate **26** and sodium stibogluconate (Pentostam^®^) **27**), pentamidine **28**, and amphotericin B **29** (Figure 4) [85]. Antimonials are still used for the treatment of any type of leishmaniasis. However, they suffer from drug toxicity such as hepatic, severe cardiac, renal, and pancreatic toxicity. Nevertheless, their use also results in a high cure rate and they are affordable. They are usually administered intravenously but are associated with adverse effects such as abdominal colic, myalgia, skin rashes etc. Pentamidine was initially available as pentamidine methanesulfonate and pentamidine isethionate salt [86]. The formulation of pentamidine methanesulfonate was terminated and presently, only pentamidine isethionate is still in use. The severe adverse effects of pentamidine include renal impairment, hypotension, and diabetes mellitus etc. [85]. Amphotericin B is the key treatment choice in cases of drug resistance to antimonials. It offers a high cure rate of 100% when administered intravenously despite its side effects requiring hospitalization for a period of one month [85]. Pentavalent antimonials are used as the first line of treatment in European countries [87,88]. Liposomal amphotericin B (AmBisome^®^) is administered by intravenous infusion and it is approved by the FDA for the treatment of visceral leishmaniasis. Miltefosine is used for the treatment of visceral, cutaneous and mucosal leishmaniasis. It is administered orally. Other drugs used for the treatment of leishmaniasis are referred to as the azoles (fluconazole, ketoconazole, itraconazole). They are administered orally. However, the use of azoles has shown mixed results. Topical formulation of paromomycin has also been reported for the treatment of leishmaniasis [87,88,89].

Artemisinin and its derivatives have also been reported to exhibit antileishmanial activity. Artemisinin has been reported to be effective against visceral leishmaniasis. In vitro studies revealed that artemisinin displayed IC_50_ values in the range of 100–120 µM against promastigote. Furthermore, artemisinin was reported to increase the production of nitrite and mRNA expression of inducible nitric oxide synthase resulting in the protection of the host and parasitic activity. In vivo studies on BALB/c mice induced with visceral leishmaniasis were administered artemisinin orally (10 mg/kg and 25 mg/kg body weight). A reduced splenic weight and parasite burden was significant [90]. Artemisinin also acts by generating free radicals within the Leishmania parasites [91]. It induced apoptotic effects on promastigotes of *Leishmania major.* Cell apoptosis is effective in eliminating the parasite. A study showed that artemisinin exhibited apoptotic effect against promastigotes of *Leishmania major.* The apoptosis and late apoptosis of promastigotes of *L. major* cultured with IC_50_ dose of artemisinin (25 μg/mL) were 25.90 and 15.33 μg/mL, respectively [92]. In another study, the percentage of apoptotic promastigotes when the concentration of artemether 10, 25 and 50 μg/mL were used on *L. major* were 2.44, 42.28 and 3.83 μg/mL, respectively [93]. The mean of the amastigotes/macrophages after adding artemether at a concentration of 5, 10, 25, 50 and 100 μg/mL after 72 h were 0.78, 0.64, 0.49, 0.30 and 0.21, respectively.

### 4.2. Drug Resistance of Antileishmanial Drugs

There are several conditions that result in drug resistance in antileishmanial drugs. The antimonials suffer from drug resistance due to incorrect use by most of the patients which expose the *Leishmania* parasites to therapeutic pressure, causing the development of tolerance, and ultimately drug resistance [94]. Other factors that can lead to the emergence of drug resistance in antimonials include the reduction of drug accumulation inside the parasite, either by increasing drug efflux or decreasing drug cellular uptake, inactivation of an active therapeutic agent, inhibition of drug activation, and amplification of genes [95]. Furthermore, the mechanism of pentavalent antimonial resistance is due to overexpression of the membrane-bound Adenosine Triphosphate (ATP)-binding cassette (ABC) transporters on the surface of the parasites [96]. The drug resistance of pentamidine in *Leishmania* parasite has been defined based on mutations in several transporters. ABC transporters have been recognized from various species of *Leishmania* and associated with resistance reported with pentamidine [97]. Aquaglyceroporin 2 (AQP2), a family member of surface channel proteins involved in the water passive transport across the cell membrane, is known as the transporter responsible for drug resistance to high pentamidine concentration in trypanosomes [97]. AQP2 mutation also leads to drug resistance of pentamidine in pathogenic parasites.

There are several studies that have been carried out to understand amphotericin B resistance. The biological characteristics of the resistant strains were compared to the wild-type parent strain and some mutations were found [98,99]. These mutations include a mutation in lanosterol 14α-demethylase, sterol biosynthesis enzyme in a *L. mexicane* cell line. There were genetic alterations in multiple AMB-resistant Leishmania lines that were also found in 2 sterol biosynthesis enzymes: sterol C5-desaturase which is essential for the generation of sterol 5(6)–7(8) double bond incorporation, and sterol C24-methyltransferase which introduces the C24-methyl group within the ergosterol side chain [99]. Generally, drug resistance can be caused by reduced amphotericin B binding to the membrane because of a changed sterol profile (loss of function of the Sterol C24-methyltransferase gene). Amphotericin B is effluxed out by the membrane-bound 1 and the remaining intracellular amphotericin B auto-oxidizes and produces reactive oxygen species [99].

## 5. Nanoparticles

Nanoparticles are drug delivery systems that are particulate dispersions or solid colloidal particles with diameters ranging between 1 to 1000 nm [100]. These nanocarriers are usually developed from polymers, lipids/proteins, metals and carbon-based materials. Examples of polymers that are used for the formulation of polymer-based nanoparticles include poly (d,l-lactic acid), poly (d,l-lactic-*co*-glycolic acid) (PLGA), polyaspartamide, polyalkylcyanoacrylates, etc. [101]. The unique properties of polymers which make them useful in drug delivery are their good compatibility and biodegradability with high stability in a biological environment [102]. Their unique properties are due to factors such as their chemical structure, the type of the functional groups in the molecule, the degree of polymerization, the method of synthesis etc. [103,104]. Drugs are loaded to polymers by encapsulation or immobilization on the polymer for drug release at the target sites [105]. The unique characteristic of the polymer-based nanoparticle is their controlled release mechanism of the loaded therapeutic agents.

Metal-based nanoparticles can be prepared from zinc oxide, iron oxide and other metallic oxides [106]. They are prepared from metals precursors. They exhibit good optoelectrical properties due to localized surface plasmon resonance characteristics. Some of them exhibit a broad absorption in the visible zone of electromagnetic spectrum such as alkali and noble metal nanoparticles. Their advanced optoelectrical properties make them suitable for diagnostic application and targeted drug delivery [107].

Lipid nanoparticles are biodegradable in the biological environment with low toxicity when compared to the polymeric nanoparticles [106]. Lipid nanoparticles are biocompatible because their conformation resembles plasma membrane lipids and human cholesterol. Examples of lipid nanoparticles are liposomes, polymeric nanoparticles and nanoemulsions. Liposomes are composed of a lipid bilayer made of anionic, cationic, or neutral phospholipids and cholesterol. It is enclosed with an interior aqueous space. Liposomes are classified based on the structure of their bilayer such as unilamellar and multilamellar vesicles [108,109]. Unilamellar vesicles have a single-lipid bilayer with a diameter of 20–250 nm diameter and they are suitable for the encapsulation of hydrophilic drugs. Multilamellar vesicles are made up of two or more lipid bilayers with a diameter of 1–5 μm. They are used for the encapsulation of molecules which are soluble in lipids. Phospholipids act as a barrier which protects the formulation from the action of pH, enzymes, and free radicals in the biological environment and physiological conditions that results in premature degradation before reaching the target cell/tissues. They have distinct properties such as good biocompatibility, low toxicity, high biodegradability, and they can be used to encapsulate hydrophilic and hydrophobic molecules [108,109]. Some formulations of liposomes are available in clinical use and some liposomal formulations are currently tested in different phases of clinical trials.

Nanoemulsions are colloidal systems with a size range of 10–1000 nm. They are characterized by an amorphous and lipophilic surface. There are three types of nanoemulsions namely: oil in water nanoemulsions; water in oil nanoemulsions and bi-continuous nanoemulsions. They exhibit good properties such as improving the bioavailability of the loaded drug, extending the action of the encapsulated bioactive agents, they are non-toxic and biocompatible, display good physical stability, they have high surface area suitable for excellent absorption, they can solubilize lipophilic drug, they mask taste and need less amount of energy [110,111].

Lipid nanoparticles are classified into two groups: nanostructured lipid carrier (NLCs) and solid lipid nanoparticles (SLNs). SLNs have particle sizes less than 1000 nm. Their use is limited by their perfect crystalline structure and low drug loading efficiency. They display drug molecules orientation between the fatty acid chains or glycerides. NLCs are the second generation of lipid-based nanocarriers and they are developed to overcome the limitations associated with SLNs making them effective systems for drug delivery [112]. Lipid nanoparticles can deliver a sustained and high level of the loaded drug in the blood plasma [113]. There are different routes of administration of lipid nanoparticles such as oral, ocular, pulmonary, cerebral and topical [114,115]. There are various modes of action utilized by these nanoparticles to target cells or tissues specifically. One of the mechanisms is through binding to certain cell surface receptors that may be overexpressed on the anticipated target cells/tissues [116]. These drug delivery systems can also diffuse into cells or tissues through passive permeability. The general functions of nanoparticles include improved aqueous solubility, their suitability for co-delivery of drugs, enhanced drug biocompatibility, and biodegradability, sustained and controlled drug release profiles, and improved drug activity [71]. The aforementioned features of nanoparticles are due to their small size and large surface area. Their small size enhances their cellular uptake. Furthermore, the surface of the nanoparticles can be manipulated by the incorporation of targeting ligands etc. for the prevention of aggregation and rapid clearance, to enhance their stability in a biological environment, for a controlled/sustained drug release mechanism and for targeted drug delivery [117].

Due to the aforementioned features of nanoparticles, they are useful for the delivery of artemisinin and its derivatives. Artemisinin and its derivatives are limited by their poor water solubility and low bioavailability. They have been reported to be effective for the treatment of parasitic diseases such as malaria, leishmaniasis and chronic disease such as cancer etc.

### 5.1. Nanoparticles Containing Artemisinins for Cancer Treatment

Tumor vasculatures are characterized by abnormal, leaky walls and large pores [118]. Their leaky walls are due to the accelerated proliferation of endothelial cells and a reduced number of pericytes. The diameter of their pore sizes ranges from 40 nm to several hundred nanometers when compared to the normal vessels which are in the range of 5–10 nm [119,120]. These large pores of tumor vasculature promote higher permeability and allow nanoparticles uptake into the tumors [118]. Nanoparticles display prolonged retention in the tumor resulting in their high concentrations in the tumor when compared to other tissues. Their uptake into the tumor tissues is via the leaky vessels by a mechanism known as the enhanced permeability and retention effect (EPR) [119]. Nanoparticles with a diameter less than 200 nm are taken up effectively into the tumor cells [121]. Their passive accumulation via the leaky tumor vasculature is via Enhanced Permeability and Retention mechanism resulting in high drug accumulation and improved treatment efficacy. EPR effect is heterogeneous and varies in different tumors and patients. Due to the aforementioned reasons, active targeting is considered an effective approach for novel nanoparticles therapeutics. Active targeting of nanoparticles to tumor cells involves the surface modification of the nanoparticles with antibodies, peptides, ligands, small molecules, etc. to promote targeting of the particles to the receptors present on the target cell/tissue. Active targeting enhances high nanoparticles accumulation in the tumor [120]. There are several reports of nanoparticles loaded with artemisinin and its derivatives for the treatment of cancer (Table 2).

#### 5.1.1. Polymer-Based Nanoparticles Loaded with Artemisinin and Derivatives with Anticancer Activity

Natesan et al. formulated polymeric magnetic nanoparticles loaded with artemisinin to target breast cancer cells using a biopolymer, chitosan [122]. The formulation was prepared by an ionic gelation technique. The Fourier Transform Infrared spectroscopy (FTIR) analysis confirmed the physicochemical properties of the nanoparticles. The average particle size was in the range of 349.3–445.9 nm, polydispersity index (PDI) of 0.908, surface charge ranging between −9.34 and −33.3 mV, and spherical shaped morphology with a smooth surface. The loading capacity and encapsulation efficiency of the nanoparticles increased as the chitosan concentration increased. The in vitro drug release showed the percentage of artemisinin release was 62.85, 65.04 and 78.78%, depending on chitosan concentration and the average particle size over a period of 48 h. The in vitro cytotoxicity activity of the free artemisinin and the drug-loaded nanoparticles on MCF-7 cells employing MTT assay further revealed the low cytotoxic effect of artemisinin-loaded nanoparticles when compared to the free artemisinin with IC_50_ values of 25.61 ± 13 µg/mL for the nanoparticles and 16.25 ± 6 µg/mL for the free artemisinin. Furthermore, in vivo studies revealed an enhanced accumulation of the nanoparticles in the 4T1 breast tumor tissues of BALB/c mice model [122]. Chen et al. prepared conjugates of artesunate incorporated to a long chain of *N*,*N*′-bis(dodecyl)-l-glutamic diamide. The formulation mediated ROS generation and targeted the mitochondria, a target for inducing cancer cell death and bypassing multi-drug resistance (MDR) mechanisms [123]. Liu et al. developed artesunate-loaded bovine serum albumin nanoparticles for mitochondrial targeting. The formulation displayed a high cytotoxic effect and a significant apoptotic effect due to the high accumulation of artesunate in the mitochondria. The drug-loaded nanoparticles induced mitochondrial-mediated cell apoptosis [124]. Mitochondria are involved in a variety of apoptotic signals. They are attacked by drugs thereby enhancing their permeability resulting in endometrial swelling of their membrane and the release of cytochrome C into the cytoplasm [125]. Liu et al. prepared transferrin-eight-arm-polyethylene glycol-dihydroartemisinin nanoparticles. The nanoparticles mean size was 147 nm with high solubility and drug loading of 10 wt % dihydroartemisinin. They also displayed prolonged circulating half-life. In vivo studies on a Lewis lung tumor bearing animal model revealed a significant growth inhibition effect of the drug-loaded nanoparticles when compared to the free drug. The PEGylated nanoparticles prolonged the circulation time of the formulation [126].

Nguyen and co-workers formulated PLGA-based nanoparticles loaded with artesunate via an oil/water emulsion evaporation technique [127]. The particle size analysis using Dynamic light scattering analysis (DLS) showed a nanometric size of approximately 170 nm and PDI of 0.2. The drug loading capacity of artesunate was 23.67%. X-ray Diffraction analysis (XRD) spectra revealed the crystalline nature of the free drug and the amorphous nature of the drug-loaded PLGA-nanoparticles confirming the successful loading of artesunate into the nanoparticles. In vitro drug release profile of PLGA-nanoparticles was a burst drug release at the initial 10 h and this release extended up to 60% and 80% for formulations containing Tween 80 and sodium lauryl sulfate, respectively. In vitro anticancer evaluation was performed on MCF-7, SCC7, and A549 cell line utilizing MTT assay after 1-day exposure of the cell lines to the drug-loaded nanoparticles. Artesunate-loaded PLGA-nanoparticles demonstrated a strong cytotoxic effect when compared to the free artesunate. The cytotoxic effect of the drug-loaded nanoparticles was significant in MCF-7 and A549. The cell viability was greater than 80% in the presence of the blank nanoparticles when compared to the control [127]. Furthermore, the drug-loaded nanoparticles decreased cell viability in a concentration-dependent manner. The nanoparticles protected artesunate from epimerization and hydrolysis. The small size of the nanoparticles enhanced their capability to cross the abnormal tumor vasculatures thereby delivering the drug to the tumor by passive diffusion via an EPR effect. Furthermore, the presence of Tween-80 on the surface of the nanoparticles decreased the efflux of the free drug by efflux pumps such as P-glycoprotein (P-gp) [127].

Li and co-workers formulated and evaluated PLGA-based nanoparticles containing dihydroartemisinin by emulsion solvent evaporation technique [128]. Dihydroartemisinin was conjugated via an ester linker to the terminal carboxyl of PLGA-PEG2000-COOH. The average particle size of the nanoparticles was approximately 145 nm with a zeta potential of −4 mV. The encapsulation efficiency and the drug loading capacity of the nanoparticles was 93% and 4.4%, respectively. The in vitro drug release studies at pH 7.4 and 37 °C was a sustained drug release pattern of dihydroartemisinin from the nanoparticles. The in vitro cytotoxicity evaluation using the MTT assay method on 3T3 and 4T1 showed a concentration-dependent mechanism. This evaluation displayed approximately 3.1-fold greater in cancerous 4T4 cells when compared to the non-cancerous 3T3 cells. Pharmacokinetics studies revealed a prolonged circulation time of the nanoparticles which was 6.2 h [128]. In addition, an in vivo antitumor efficacy study showed a reasonably delayed growth of the tumor when compared to the control groups. These nanoparticles stimulated tumor cell apoptosis when compared to the nanoparticles not loaded with drugs. Factors such as good colloidal stability due to the steric PEG2000 shell, prolonged blood circulation in vivo, specific distribution in the tumor by the passive EPR effect and elevated cellular internalization in the tumor cells influenced the significant antitumor effect of the nanoparticles. Ma et al. formulated biodegradable PLGA-nanoparticles for the combination of dihydroartemisinin and doxorubicin. The cellular uptake and cell viability studies showed that the nanoparticles increased the cell internalization of the drugs, thus improving the cytotoxicity due to the high entrapment efficiency of doxorubicin and dihydroartemisinin [129].

#### 5.1.2. Lipid-Based Nanoparticles Containing Artemisinin and Derivatives with Anticancer Activity

Wang et al. synthesized lipid nanoparticles by the solvent-emulsification method for co-delivery of dihydroartemisinin and sorafenib for liver cancer targeting using cholesteryl oleatea and triolein lipids [130]. Dynamic light scattering (DLS) analysis revealed a mean particle size of 115.4 ± 1.25 nm, narrow PDI of approximately 0.112 and a surface charge in the range of (−23 mV)–(−25 mV). TEM images of the formulation showed spherical shaped-like particles without any indication of aggregation. The in vitro drug release profile at pH 7.4 and pH 5.0 displayed a release of less than 20% of drugs from the nanoparticles within 1 day and less than 40% drug release over a period of 60 h. The targeting efficiency studies of lipid nanoparticles using HepG2 liver cancer cells showed that the nanoparticles accumulated in the tumor cells more effectively when compared to the free drugs. The dual-drug loaded lipid nanoparticles showed in vitro synergistic anticancer activity which was characterized by reduced cell viability of HepG2 cells when compared to the free drugs. Dihydroartemisinin induced the generation of ROS when combined with sorafenib and displayed a synergistic anticancer effect in HepG2 cancer cells. In vivo antitumor study displayed a significant antitumor effect with delayed tumor growth. The cleavage of the endoperoxide bridge in dihydroartemisinin structure releases oxygen free radicals thereby increasing the ROS level in the cancer cells. The increase in the ROS level resulted in irreparable oxidative destruction to the lipids and intracellular DNA resulting in cell death [130].

Zhang et al. reported dimeric artemisinin piperazine lipid nanoparticles with a pH-dependent aqueous solubility prepared from egg phosphatidylcholine. The diameter of the nanoparticles was approximately 80 nm. The drug loaded nanoparticles were effective against human breast cancer cell lines and non-toxic on non-tumorigenic cells. Furthermore, in vivo studies on MDA-MB-231 mouse xenograft model, revealed that the formulations were more effective when compared to paclitaxel solution. The cellular uptake of the nanoparticles was via endocytosis [131]. Zhang et al. also reported that the liposomal nanoparticles released the loaded artemisinin at acidic pH of the solid tumors. A down regulated anti-apoptotic protein, survivin, and cyclin D1 were observed in the breast cancer cell lines at low concentrations of the formulation. A down regulated oncogenic protein HER2 and HER3 were observed in a HER2+ cell line with a reduction in the wild type epidermal growth factor receptor (EGFR or HER1) in a triple negative breast cancer cell line [132].

Another type of lipid nanoparticles which has been designed for the delivery of artemisinin and its derivatives is liposomes. Li et al. developed functional targeting paclitaxel plus artemether liposomes for the treatment of invasive brain glioma, a lethal type of cancer. The complete removal of the tumor surgically is impossible and the poor uptake of therapeutic drugs across the Blood-Brain Barrier (BBB) makes it challenging to treat brain cancer. The liposomes were developed by tailoring a mannose-vitamin E derivative conjugate and a dequalinium-lipid derivative conjugate. The dequalinium-lipid derivative conjugate is a cationic amphiphilic phospholipid composed of delocalized cationic charge that can cross the BBB and target cancer cells. Its incorporation into the liposomes enhanced the circulation time of the formulation. It also makes the formulation stable. Paclitaxel combination with artemether liposome formulation displayed a significant inhibitory and apoptosis-inducing effects against brain cancer cells [133]. In vivo studies in intracranial glioma tumor bearing animal models, revealed a 30% tumor inhibition at day 16 for the functional targeting liposome formulation loaded with both drugs when compared to paclitaxel and artemether liposomes which was 74%. The cytotoxic effect of the formulation showed that the liposomal formulation was effective against invasive brain gliomas via the induction of apoptosis and necrosis. The transport capabilities of the formulation across the BBB was significant [133].

Righeschi et al. developed unilamellar vesicles loaded with artemisinin from cholesterol, P90G, and DSPE-PEG2000-COOH. The liposomes were conjugated to the lipid linker via the carboxyl residue of DSPE-PEG-COOH. Transferrin was conjugated to the liposome and the cellular uptake of the formulation was significant. The IC_50_ value of drug-loaded liposomes formulation was 69 µM when compared to the free artemisinin which was 127 µM on MCF-7 breast cancer cells suggesting that transferrin played a crucial role in the efficacy of the formulation in vitro [134]. Leto et al. also reported similar findings in which artemisinin was loaded into transferrin-conjugated liposomes. The cell uptake and cytotoxicity studies of the formulation in HCT-8 cell lines confirmed an enhanced uptake of the formulation due to the presence of iron ions [135]. Kang et al. prepared tumor-targeting mannosylated liposomes loaded with doxorubicin and dihydroartemisinin. The mean diameter of the formulation was 158.8 nm with a zeta potential of −15.8 mV. In vivo studies on a subcutaneous HCT8/ADR tumor xenograft model showed that the administration of the drug loaded liposomes inhibited tumor inhibition at a rate of 88% when compared to free doxorubicin and a combination of doxorubicin and dihydroartemisinin which were 47.46% and 70.54%, respectively. The mechanisms of the formulation were based on preferential nuclear accumulation of the loaded drugs, downregulation of Bcl-xl, increased cancer cell apoptosis, and the induction of autophagy [136].

Righeschi et al. loaded dihydroartemisinin into conventional liposomes (P90G and cholesterol) and stealth liposomes (P90G; cholesterol and PE 18:0/18:0 PEG 2000). The cellular uptake of the conventional liposomes was higher than the stealth liposomes indicating that the hydrophilic steric barrier of PEG molecules in the stealth liposomes reduced its cellular uptake. The liposomes revealed the absence of toxicity [137]. Chen et al. reported liposomes encapsulated with artemether with sonodynamic anticancer activity. The diameter of the liposomes was 150 nm. The liposomal formulation intracellular uptake was significant with a high generation of ROS in HepG2 cells and it improved the killing efficiency of the tumor cells by ultrasound radiation. The combination of artemether with the liposomes enhanced the anticancer effect significantly. The ultrasound promoted rapid drug release from the liposomal formulation due to the formation of pore-like defects in the liposomes membrane [138]. Tian et al. developed liposomes loaded with artemether for intravenous delivery for the treatment of metastatic tumors. The average particle size of the formulation was 187.3 nm. The in vitro drug release of artemether from the formulation was sustained. In vivo studies, in animal models administered the formulation intravenously, revealed a growth inhibition rate which was 1.54 times higher when compared to the animal model administered the free drug solution. The area under the plasma drug concentration-time curve (AUC) of the drug-loaded formulation was 3.11-fold when compared to the free drug solution [139].

Emulsions loaded with a derivative of artemisinin, dihydroartemisinin was reported by Wang et al. Oil-in-water injectable emulsion formulation of dihydroartemisinin was developed. In vivo studies in tumor-bearing mice with transplanted murine hepatic H22 cells administered the formulation intravenously displayed a high tumor growth inhibition of 51.8% and the smallest tumor volumes with reduced side effects when compared to the free dihydroartemisinin. The half-life of the drug was extended significantly in the animals treated with the drug-loaded emulsions when compared to the free drug solution [140]. These findings revealed the efficacy of emulsions of artemisinin in the treatment of cancer. However, there is a pressing need for the development and studies on more emulsions of artemisinin for the treatment of cancer.

#### 5.1.3. Metal-Based Nanoparticles Containing Artemisinin and Derivatives with Anticancer Activity

Wang and co-workers synthesized metal-based nanoparticles for dual-drug delivery of dihydroartemisinin using Fe(III) ions, Fe_3_O_4_ as a carrier [141]. Transmission electron microscope (TEM) and scanning electron microscope (SEM) images of metallic nanoparticles displayed a uniform particle size of approximately 110 nm and a monodispersed sphere-shaped morphology. The drug loading capacity of dihydroartemisinin in the nanoparticles was 804.9 mg/g with a loading efficiency of 80.5%. The in vitro anticancer efficiency of the free drug and drug-loaded nanoparticles was evaluated using MTT assays in HeLa and A549 cancer cell lines. The metallic nanoparticles were non-toxic with cell viability higher than 101.4% (for A549 cells) and 81.8% (for HeLa cells) though the concentration was as high as 100 mg/mL which indicated the good biocompatibility of the nanoparticles [141]. Hou et al. prepared transferrin modified hollow mesoporous CuS nanoparticles loaded with artesunate. The uptake of the formulation in MCF-7 cells was via Tf-mediated endocytosis converting near infrared light to heat for photothermal therapy with the generation of high levels of reactive oxygen species (ROS). In vivo studies on tumor-bearing mice via peritumoral injection of the formulation under near infrared laser irradiation revealed a significant inhibition rate of 74.8%. The administration of the formulation resulted in a significant drug accumulation in the tumor revealing good tumor targeting ability and retention effect when compared to the formulation without the targeting moiety. The release mechanism of the drug from the liposome was sustained with a detection of the drug 96 h after administration [142]. Hollow mesoporous CuS nanoparticles are reported to be intelligent delivery systems due to their uniform pore structure and high surface area suitable for a high rate of drug loading. They also generated cytotoxic reactive oxygen species under near infrared irradiation making it a potential system for enhanced antitumor targeting [143,144].

Chen and co-workers prepared multifunctional mesoporous Fe_3_O_4_ or Ag-based nanoparticles loaded with artemisinin and Fe^2+^ for synergistic tumor growth inhibition. FTIR, XRD, TEM and UV-vis spectroscopy confirmed the physicochemical properties of the nanoparticles. These nanoparticles’ drug loading content and encapsulation efficiency was 303 mg/g and 48.8%, respectively. The cellular uptake studies of the nanoparticles on MCF-7 breast cancer cells showed that the metal-based nanoparticles were freely taken up into the cancer cells. The tumor growth inhibition effects of the nanoparticles employing MTT assay in HeLa cells at various concentrations showed a dose-dependent cell viability reduction when compared to the free drugs [145]. Wang et al. prepared metal-based magnetic nanoparticles loaded with the prodrug artesunate using Fe_3_O_4_ carriers. The cell growth inhibition studies showed that there was a decreased cell viability of K562 cancer cells when treated with artesunate-loaded nanoparticles compared to the free artesunate [146].

Guo et al. loaded dihydroartemisinin into magnetic nanoparticles prepared from iron oxide precursor. The nanoparticles increased the amount of reactive oxygen species and exhibited a significant killing effect on breast cancer cells, MCF-7 cells. The nanoparticles also displayed a high toxic effect to drug-resistant breast cancer cells, MCF-7/ADR cells revealing its ability to overcome multidrug resistance. The nanoparticles produced ferrous ions in an acidic condition of the tumor microenvironment which induced the artemisinin derivative to produce a large amount of ROS resulting in cell death. Furthermore, the nanoparticles exhibited a significant inhibitory effect on aggressive breast cancer cell lines (MDA-MB-231 and MDA-MB-453 cells) [147].

#### 5.1.4. Carbon-Based Nanoparticles Loaded with Artemisinin and Derivatives as Anticancer Therapeutics

Carbon-based biomaterials have been used to develop drug delivery systems due to their unique features such as large surface area, chemical stability, good mechanical properties etc. Liu et al. formulated graphene oxide-based nanoparticles for dual-drug loading of dihydroartemisinin and transferrin for tumor targeting [148]. Transferrin and dihydroartemisinin were modified on the surface of graphene oxide (GO) nanoparticles for tumor therapy. Atomic Flame microscopy (AFM) and the TEM images of the nanoparticles displayed a particle size range of 100–200 nm. The in vitro cytotoxicity studies of the dual-drug loaded nanoparticles revealed that their cytotoxic effect was dose-dependent with a synergistic effect when compared to single-drug loaded nanoparticles. The metabolism and biodistribution studies of the nanoparticles in vivo using mice infected with EMT6 tumors showed a significant accumulation of the graphene oxide-based nanoparticles in the tumor. Furthermore, the in vivo antitumor evaluation indicated a complete tumor cure when the dual-drug loaded nanoparticles were administered within a month when compared to the free drug that showed limited tumor growth delay. The decoration of transferrin on the GO nanoparticle enhanced the targeting efficacy of the nanoparticle to the tumor cells with overexpressed transferrin receptor thereby increasing iron ion that interacts with dihydroartemisinin. This interaction results in a high intracellular ROS and enhanced the drug cytotoxicity [148].

Zhang et al. developed hyaluronic acid-derivatized multi-walled carbon nanotubes modified with transferrin as targeting ligand and loaded with artemisinin. The drug-loaded formulation displayed a synergistic antitumor effect when compared to the free drug in vitro in MCF-7 cells and in vivo in tumor-bearing murine model. Increased intracellular drug uptake was significant with a high inhibition effect [149]. Zhang et al. also grafted hyaluronic acid to fullerene which was then combined with transferrin for multi-functional drug delivery. Artesunate was adsorbed to the aforementioned biomaterial with a high drug loading efficacy of 162.4%. The enhanced antitumor efficacy of drug adsorbed nanoparticles in MCF-7 cells in vitro and in a tumor-bearing murine model in vivo was due to the increased cellular uptake of artesunate into the tumor. The tumor inhibition rate was 64.74% when compared to the free artesunate which was 20.14%. The retention of artesunate concentration in the tumor was 131.06 µg/g when compared to the free drug which was 11.13 µg/g indicating that the transferrin enhanced the drug accumulation into the tumor [150].

### 5.2. Nanoparticles Containing Artemisinins for Malaria Treatment

The use of nanoparticles as drug delivery systems promotes protection against premature extracellular degradation, improves targeted drug delivery, lowers the frequency of drug administration and improves the pharmacokinetic profiles of the drug [151]. The unique properties of the nanoparticles are their ability to be retained in the blood for an extended period resulting in improved interaction with infected red blood cells and parasite membranes [152]. The strategies for targeting antimalarial drugs to the infected erythrocytes and hepatocytes using nanoparticles is via passive and active targeting. Passive targeting is achieved using conventional nanoparticles such as liposomes, polymeric nanoparticles etc. Active targeting is achieved by using nanoparticles with a surface modified with ligands such as proteins, antibodies etc. [153,154]. Passive targeting is not exploited in malaria by the intravenous route because the red blood cells are not endocytically and phagocytically active. The exposure of phagocytes to an overload of nanoparticles can result in an initial blockage of the phagocytic uptake followed by a subsequent rise in the macrophage capacity [155]. This effect can lower the quick action of antimalarial drugs loaded in a nanoparticle. However, these strategies produce a depot that promotes slow drug intake into the blood, resulting in an alteration in the pharmacokinetic profile of antimalarial drugs with a short half-life.

The modification of the surface of nanoparticles with hydrophilic polymers can result in delayed phagocytosis thereby extending the drug half-life in the blood with a regulated biodistribution and the pharmacokinetic profile of the drug [154]. Passive targeting to mononuclear phagocyte system can be employed in malaria treatment. However, long-circulating nanoparticles are suitable for intravenous delivery due to enhanced contact with the red blood cells. In active targeting of bioactive agents using nanoparticles, the surface of the nanoparticles is modified with a cell-specific ligand to promote targeted uptake of the drug in the target cell/tissue [154,156]. This strategy is useful for parenteral administration for the management of cerebral malaria. In malaria, the main targets are erythrocytes and the hepatocytes in the blood and liver, respectively.

#### 5.2.1. Polymer-Based Nanoparticles Loaded with Artemisinin and Derivatives with Antimalarial Activity

Jain et al. formulated and studied PLGA-based nanoparticles containing artemisinin via nano-precipitation methods [157]. The average particle size of the nanoparticles was less than 200 nm with polymer index (PI) of 0.45, depending on the amount of PLGA. The encapsulation efficiency of the drug was in the range of 73.5%–97.8%. The SEM images of the polymeric nanoparticles loaded with artemisinin displayed a spherically-shape morphology with a diameter of 100 nm. The in vitro drug release studies of drug-loaded PLGA nanoparticles exhibited a burst release of 20% of artemisinin in the first 10 min. Polymeric nanoparticles loaded with artemisinin displayed a 95.5% release of the drug in 45 min. The cumulative drug release of the free artemisinin was 74.2% in an hour resulting from its low solubility [157]. The drug release studies followed the Korsmeyer Peppas drug release model. Oyeyemi et al. formulated polymeric nanoparticles for co-encapsulation of artesunate and curcumin via solvent evaporation using PLGA carriers. The particle size analysis of the nanoparticles exhibited particle size, zeta potential, and PDI of 251.1 ± 12.6 nm, −19.1 ± 4.9 mV, and 0.121 ± 0.06, respectively. The total co-entrapped drugs in the nanoparticles was 22.3%. The in vitro drug release profiles of the nanoparticles showed a controlled and sustained drug release over a period of one week. The in vivo antimalarial studies on *P. berghei* showed high suppression rate of 79% at day 5 when compared to 72.5% at day 8 at a dosage of 5 mg/kg drug-loaded nanoparticles [158].

Yaméogo and co-workers formulated self-assembled biotransesterified cyclodextrin nanoparticles loaded with artemisinin via a solvent displacement technique. These nanoparticles demonstrated PI value in the range of 0.03–0.06 with particle sizes in the range of 90–190 nm. The in vitro drug release profile was sustained and controlled from the cyclodextrin nanoparticles. Furthermore, in vitro antiplasmodial activity evaluation revealed growth inhibition of *P. falciparum* both on susceptible 3D7 and multi-resistant K1 strains with IC_50_ values of 7.0 and 2.8 ng/mL, respectively [159]. In addition, Yaméogo et al. prepared similar nanocarriers using amphiphilic γ-cyclodextrin for intravenous administration of artemisinin. The nanoparticles exhibited a mean particle size and negative zeta potential of 92 nm and −20 to −30 mV, respectively. The pharmacokinetic study of the nanoparticles was performed to evaluate any improvement in the artemisinin bioavailability from the amphiphilic γ-cyclodextrin nanoparticles and it displayed a 4.00- and 6.25-fold plasma half-life when compared to the free artemisinin. The intravenous administration of artemisinin-loaded nanoparticles resulted in a longer elimination half-life when compared to the free artemisinin indicating that they are potential therapeutics for the treatment of severe malaria [160].

Chadha et al. assembled chitosan/lecithin nanoparticles loaded with artesunate and artemisinin complexed with β-cyclodextrin for enhanced antimalarial activity of the loaded drugs. The particle size distribution of the formulation was less than 300 nm with a drug entrapment efficiency of 90% for the nanoparticles loaded with 100 mg of artesunate. In vitro drug release behavior of the nanoparticles was pH-dependent. In vivo studies in *Plasmodium berghei* mice by oral administration of the formulation revealed less mean percent parasitemia showing the efficacy of the formulation for the treatment of malaria [161].

#### 5.2.2. Lipid-Based Nanoparticles Loaded with Artemisinin and Derivatives with Antimalarial Activity

Wadzanayi et al. prepared and evaluated solid lipid nanoparticles loaded with artesunate via a microemulsion dilution method using glyceryl monostearate as the carrier [162]. The drug loading and encapsulation efficiency of artesunate in lipid nanoparticles were 2.44% and 51.7%, respectively. The particle size analysis of the solid lipid nanoparticles showed mean particle size and PDI of 1109 nm and 0.082, respectively with a zeta potential of −20.7 mV. The in vitro drug release studies of the drug loaded nanoparticles at pH 6.8 revealed a lower release of approximate 29.95% of artesunate in the first 15 min and a gradual increase after 4 h to a maximum of 63.64%. The drug release mechanism at pH 1.2 was sustained with 46.08% drug release in 5 h. The intestinal permeability studies of the lipid nanoparticles showed an enhanced permeability of artesunate which can result in an improved rate of artesunate absorption leading to an improved therapeutic activity [162].

Omwoyo et al. formulated and characterized solid lipid nanoparticles loaded with dihydroartemisinin via single-emulsion solvent evaporation method utilizing steric acid. The steric acid-based nanoparticles showed a significant drug encapsulation efficiency percentage of 62.3%. The particle size analysis of the dihydroartemisinin-loaded nanoparticles exhibited particle size, PDI, and zeta potential of 240.7 ± 2.4 nm, 0.16 ± 0.02, and +17.0 ± 2.4, respectively. The in vitro drug release studies of the nanoparticles at 37 °C demonstrated a robust release of 23% of dihydroartemisinin. The in vitro antiplasmodial activity of nanoparticles showed an IC_50_ of 2.35 ng/mL for dihydroartemisinin-loaded lipid nanoparticles and 0.95 ng/mL for the free drug, dihydroartemisinin. The in vivo studies showed a good parasite suppression of 99.63% for dihydroartemisinin-loaded lipid nanoparticles when compared to 73.12% for the free dihydroartemisinin [163].

Aditya and co-workers formulated lipid-based nanoparticles employing soybean oil (liquid lipid) and glyceryl trimyristate (as solid lipid) loaded with artemether, an artemisinin derivative by a modified thin-film hydration technique [164]. The characterization of the nanoparticles showed that the encapsulation efficiency, TEM, average particle size and surface charge of 97%, spherical-like shaped morphology, 120 nm and −38 mV, respectively. The in vitro drug release profile of artemether from the nanoparticles was a burst drug release in the initial stage followed by a sustained drug release pattern. The in vitro hemolytic toxicity evaluation of lipid nanoparticles displayed hemolysis in the range of 7.7%–8.7%. The in vivo antimalarial efficacy of artemether-loaded lipid nanoparticles was evaluated using mice infected by *P. berghei* model. This evaluation showed good *Plasmodium* parasite suppression with a long survival period of the mice when administered the drug-loaded nanoparticles compared to the free drug [164].

Boateng-Marfo et al. developed human serum albumin-bound artemether nanoparticles prepared by emulsification and desolvation approaches for intravenous administration. The nanoparticles size was less than 50 nm. The concentration of the human serum albumin used influenced the drug concentration. The nanoparticles displayed significantly enhanced solubility when compared to the free drug. In vitro hemolysis studies revealed a decreased hemolysis caused by the formulation prepared by emulsification which was 7% and by desolvation, it was 3.68%, demonstrating a significant reduction in the hemolytic effect of artemether. These findings showed the protective effect of human serum albumin on erythrocytes [165]. The emulsification method enhanced high drug entrapment and required less organic solvent, less water and less energy. Emulsification was reported to be the preferred approach [165]. Attama et al. prepared solid lipid nanoparticles encapsulated with two antimalarial drugs, artemether and lumefantrine. The particle sizes of the nanoparticles were in the range of 150–500 nm. The crystal properties of artemether and lumefantrine influenced their solubility in the lipid matrix and their loading in the nanoparticles. In vitro studies using Caco-2 cells showed the ability of the lipid nanoparticles to deliver the drug to the gastrointestinal tract. In vivo studies indicated a high clearance of parasitemia with minimal side effects [166].

Liposomes have been developed and loaded with artemisinin and its derivatives. Isacchi et al. prepared artemisinin-loaded conventional and polyethylene glycol (PEGylated) liposomes. The encapsulation efficacy of the liposomal formulation was more than 70%. The mean particle size diameter was in the range of 130–140 nm. Intraperitoneal administration of both formulations in a healthy animal model displayed extended blood-circulation time and was detectable after 3 and 24 h of administration of the conventional and PEGylated liposomes, respectively. However, the free artemisinin was not detected 1 h after administration. AUC_0–24h_ values of the liposomal formulations were 6-fold greater when compared to the free artemisinin. Furthermore, the half-life of the artemisinin was enhanced by more than a 5-fold increase when incorporated into the liposomes. The unique features of the liposomal formulations suggest that they are suitable for the treatment of parasitic diseases such as malaria etc. [167]. Ismail et al. prepared dimeric artesunate phospholipid-based liposomes by esterification of artesunate and glycerophosphorylcholine. The liposomes were characterized by multilamellar vesicle structures with an average hydrodynamic diameter of 190 nm and a zeta potential of −20.35 mV. The drug loading capacity of the liposomal formulation was 77.6%. The liposomes were stable under neutral physiological conditions. However, the drug release was rapid in simulated acidic physiological conditions. In vivo studies revealed that the liposomes displayed longer retention half-life in the bloodstream resulting from the particle size and zeta potential of the drug-loaded liposomes. The drug-loaded liposomes resulted in enhanced parasites killing in *P. berghei*-infected mice in vivo with delayed recrudescence and improved survival when compared to the free drug. In vitro studies revealed the IC_50_ of 0.39 nM for the drug-loaded liposomes when compared to the free drug with IC_50_ of 5.17 nM showing excellent in vitro antiplasmodial activities. There was an absence of hemolysis of the erythrocytes [168].

Waknine-Grinberg et al. designed liposomes-encapsulated water-soluble glucocorticoid prodrugs in combination with artemisone for the treatment of cerebral malaria. Administration of artemisone after treatment with the liposomes formulation resulted in a complete cure. The combination resulted in a reduced level of cerebral inflammation, hemorrhage and edema. The liposomal formulation displayed significant accumulation in the brains of the sick mice when compared to the healthy mice indicating the formulation capability to disrupt the blood-brain barrier. The combination was well-tolerated and useful for the elimination of parasites and the prevention of cognitive damage in the long-term [169]. Aditya et al. combined α/β arteether with curcuminoids-loaded liposomes prepared from phosphatidylcholine by the thin-film hydration method. In vivo studies in *Plasmodium berghei* infected mice showed that the combination of curcuminoids-loaded liposomes (40 mg/kg body weight) and α/β arteether (30 mg/kg body weight) cured the infected mice and also prevented recrudescence significantly. Arteether decreased the parasitemia rapidly and the remaining parasites were cleared by the slow release of curcuminoids from the liposomal formulation [170].

Emulsions have also been designed for the delivery of artemisinin and its derivatives. Kakaran et al. prepared a nanosuspension of artemisinin for enhanced drug dissolution [171]. The crystallinity of the nanoparticles formulation decreased with an increase in drug concentration. The particle diameters were in the range of 100–360 nm. The dissolution of the drug-loaded nanoparticles was enhanced when compared to the free artesunate [171]. Ma et al. prepared lipid emulsions loaded with lumefantrine, artemether, or lumefantrin in combination with artemether for parenteral administration. In vivo studies in *Plasmodium berghei*-infected mice revealed a decrease in the parasitemia levels after 3 days with a parasitemia inhibition rate of 90% at doses of 0.32 and 0.27 mg/kg, respectively for lumefantrine lipid emulsion and artemether-lumefantrine lipid emulsion. An antimalarial effect was observed for 30 days after the administration of the formulation [172]. Ibrahim et al. prepared a nanoformulation of human serum albumin-bound artemisinin for intravenous injection and for the targeting of infected erythrocytes. In vivo studies in *Plasmodium falciparum*-infected humanized mice revealed 96% parasitemia inhibition at 10 mg/kg/day. It also prolonged the mean survival time with no recrudescence. The zeta potential of the nanoparticles was −43.8 mV and attributed to their stability by preventing particles aggregation. The good stability of the nanoformulation prevented it from premature degradation. Albumin promoted passive targeting to the malaria parasite-infected erythrocytes [173].

Yang et al. developed lipid-based emulsions for intravenous co-delivery of artemether and lumefantrine which was prepared by a high-speed shear and a high-pressure homogenization technique. The uniform particle size distribution was 220 nm with an encapsulation efficiency of 99%. The low hemolysis of the lipid emulsion is attributed to the high encapsulation efficiency of the formulation revealing its suitability for intravenous administration. After intravenous administration, artemether was rapidly eliminated with a short half-life (t1/2β, 0.11 h). However, the high maximum concentrations (C_max_) of artemether and lumefantrine were 452.86 and 2844.15 ng/mL, respectively, in the lipid emulsion group when compared to the drug solution group which were 37.92 and 918.94 ng/mL [174]. Memvanga et al. prepared lipid-based drug delivery systems loaded with curcumin (30 mg/g). The particle sizes of the nanoparticles were in the range of 30–40 nm. In *Plasmodium berghei*-infected mice, the formulation (80–100 mg/kg) was combined with lipid-based nanoparticles loaded with β-arteether (12 mg/kg) resulting in an increased survival rate and significantly delayed recrudescence. The improved drug solubilization and uptake across Caco-2 monolayers increased the antimalarial efficacy of the formulation [175]. Dwivedi et al. reported nanoemulsions loaded with arteether and prepared by a high-pressure homogenization technique. The maximum drug loading was 93% with a particle size of 156 nm and zeta potential of −23.3 ± 3.4 mV. The drug release of the formulation was sustained with 62% drug release over a period of 12 h. In vivo studies, when administered orally to multi-drug resistance *P. yoelii nigeriensis*-induced swiss mice, showed significantly improved bioavailability of arteether with AUC_0–_*_t_* 1988.411 ± 119.66 h ng/mL when compared to the free drug solution in groundnut oil which was AUC_0–_*_t_* 671.852 ± 187.05 h ng/mL. Furthermore, the *C*_max_ of formulation was 1506 ± 161.22 ng/mL which was higher when compared to arteether in groundnut oil which was 175.2 ± 16.54 ng/mL. In vivo studies further revealed a significant antimalarial efficacy of 80% cure rate at 12.5 mg/kg over a period of 5 days when compared to 30% cure rate of arteether in ground nut oil at the same daily dose [176]. Memvanga and Préat reported nanoemulsions of β-arteether for oral administration. The average particle size was in the range of 80–250 nm. The formulation was not toxic on Caco-2 intestinal cells in vitro. In vivo studies using a mouse model infected with *Plasmodium berghei* administered a daily dose of 24 mg/kg over a period of 4 days orally resulting in a 100% cure for more than 45 days. The cure rate of the formulation administered orally was comparable to the intramuscular administration of the oily solution of arteether. However, the cure rate in the mice administered an oily solution of β-arteether given orally at the same dose was lower. The formulation was reported to be safe, cost-effective and potentially, patient-friendly [177]. Parashar et al. developed artemether and lumefantrine co-loaded injectable nanostructured lipid formulation. The particle size diameter was approximately 145 nm with a zeta potential of −66 mV and encapsulation efficiency of 84% and 79% for artemether and lumefantrine, respectively. The in vitro drug release profile of the formulation was a biphasic release pattern with a release of 63% artemether and 45% of lumefantrine over a time period of 30 h. In vivo studies on animal model, *Plasmodium berghei-*infected mice showed better antimalarial activity with respect to parasitemia progression and survival period [178].

#### 5.2.3. Metal-Based Nanoparticles Loaded with Artemisinin and Derivatives with Antimalarial Activity

Kannan et al. prepared and evaluated metal-based nanoparticles loaded with artesunate using iron oxide carriers [179]. TEM and SEM images of the nanoparticles displayed an average particle size of 10 nm and sphere-like morphology, respectively. Energy-dispersive X-ray spectroscopy (EDS) confirmed the presence of the drug due to structurally rich carbon and nitrogen percentages together with the iron substantial percentage. The intracellular uptake studies of iron oxide nanoparticles using infected red blood cells (RBCs) demonstrated a significant cellular uptake of artesunate-loaded nanoparticles by the infected RBCs. The nanoparticles enhanced artesunate activity against plasmodium parasite growth with a constant effect in the range of 20–200 µg/mL of iron oxide nanoparticles, with higher cellular compatibility and negligible cytotoxicity. These studies showed that nanoparticles loaded with artesunate reduced the growth of the parasite in a dose-dependent manner. Furthermore, the in vitro studies of the nanoparticles showed that the antimalarial activity of artesunate improved 5-fold against *P. falciparum* [179].

### 5.3. Nanoparticles Containing Artemisinins for Leishmaniasis Treatment

The application of nano-based drug delivery systems for the development of antileishmanial therapies is a pressing need for enhanced pharmacokinetic properties and decreased drug toxicity effects. Macrophages phagocytose the nanoparticles resulting in target specific delivery for *Leishmania* inside macrophages [180,181]. Polymeric nanoparticles have been used as passive drug delivery systems because of their enhanced efficacy of the loaded drug. Furthermore, nanoencapsulation of antileishmanial agents improves their bioavailability resulting in the reduction of the parasite burden in macrophages. However, there are few reports on the development of nanoparticles loaded with artemisinin and derivatives evaluated as antileishmanial agents.

#### 5.3.1. Polymer-Based Nanoparticles Loaded with Artemisinin and Derivatives with Anti-Leishmanial Activity

Want and co-workers formulated and evaluated PLGA-based nanoparticles encapsulated with artemisinin by nanoprecipitation method [182]. The particle analysis of the drug-loaded nanoparticles using DLS exhibited an average particle size of 255 ± 8.03 nm and PDI value of 0.10 ± 0.015. The zeta potential of the PLGA-based nanoparticles was 9.07 ± 0.69 mV, showing good stability. The SEM and AFM images displayed a sphere-like shape and particle size of 221 ± 14 nm. The entrapment efficiency and drug loading content were 68.48 ± 1.97 and 28.03 ± 1.14, respectively showing that most of the drug was loaded in the polymeric nanoparticles. The % yield of PLGA-based nanoparticles was 22.13 ± 0.64. The in vitro drug release profile of artemisinin from the nanoparticles at pH 5.5 and 7.4 displayed a biphasic pattern consisting of a first burst drug release during the first day followed by a sustained drug release for 4 days. The ex vivo antileishmanial studies showed that PLGA-based nanoparticles greatly inhibited the intracellular amastigotes growth when compared to the plain artemisinin whereby the unloaded nanoparticles did not display any antileishmanial efficacy [182]. Want et al. also prepared and evaluated polymeric nanoparticles loaded with artemisinin via a solvent displacement technique using PLGA nanocarriers. The PLGA nanoparticles displayed a spherical shape and particles size diameter of 220 nm. The encapsulation efficiency and drug loading were 69.0% and 29.2%, respectively. These nanoparticles showed significant parasite burden reduction of *L. donovani* infection 7 days after the treatment [183].

#### 5.3.2. Lipid-Based Nanoparticles Loaded with Artemisinin and Derivatives with Anti-Leishmanial Activity

Want et al. reported the nanoliposomal formulation of artemisinin. The mean particle diameter was 83 nm with a zeta potential of −27.4 ± 5.7 mV and polydispersity index of 0.2. In vivo studies in healthy BALB/c mice revealed significantly reduced intracellular infection of *Leishmania donovani* amastigotes and the number of infected macrophages ex vivo with an IC_50_ of 6.0 ± 1.4 µg/mL and 5.1 ± 0.9 µg/mL, respectively. The formulation displayed a percentage inhibition of 82% in the liver and 77.6% in the spleen at the highest dosage of 20 mg/kg body weight [184].

## 6. Conclusions

This review article reports the therapeutic outcomes of nanoparticles loaded with artemisinin and its derivatives for the treatment of cancer, malaria, and leishmaniasis. The drug-loaded nanoparticles displayed promising biological results revealing that they are potential therapeutics for the eradication of the aforementioned diseases. The nanoparticles exhibited good cytotoxic effects against cancer cell lines when compared to free artemisinin. They also exhibited good antiparasitic efficacy on neglected parasitic tropical diseases (malaria and leishmaniasis). These nanoparticles exhibited reduced drug toxicity, enhanced the water solubility and bioavailability of the loaded drug. The only problem is that some of these nanoparticles have not reached clinical trials because of the high cost, low drug loading, and toxicity caused by the preparation strategy used.

Currently, there are very few reports on nanoparticles with antileishmanial activity indicating there is a need for more research in this field. There is an urgent need for researchers to develop these nanoparticles to reach clinical trials.

## Figures and Tables

**Table 1:** Artemisinin and derivatives anticancer mode of action on different cancers in vitro and in vivo.

Artemisinin and Derivatives	Cancer	Mode of Action	Ref.
Artemisone and Atremisinin	Breast (MCF-7) and colon (HCT116 and SW480)	Growth arrest, induction of apoptosis and blockage of the cell cycle. Decrease in the levels of its regulatory proteins CDK4 and cyclin D1	[48]
Arteether	4T1 cell line	Reduction of the cell growth of 4T1 cell line in a dose-dependent manner	[49]
Artemether	Gastric cancer cell lines (PG100)	Induced genotoxic and cytotoxic effects in the gastric cancer cell line.	[50]
Dihydroartemisinin	Pancreas cancer cell lines (PANC-1 and BxPC-3)	Up-regulation of intracellular perforin, granzyme B expression and IFN-γ production	[51]
		The production of reactive oxygen species, the modulation of apoptosis-related proteins and the induction of death receptor 5	[52]
Artesunate, dihydroartemisinin	Osteosarcoma (MG63 and 148B)	Inhibition of the growth of human osteosarcoma cells in vitro	[53]
Artemisinin		Inhibition of angiogenesis by regulating the p38 MAPK/CREB/TSP-1 signaling pathway in vivo	[54]
Artemisinin, artesunate, and dihydroartemisinin	Leukemia (MV4-11, MOLM-13, or ML-2)	Induced reactive oxygen species (ROS)-mediated apoptosis.	[55]
Artemisinin	Neuroblastoma (SK-N-AS, SK-N-DZ and SHEP1)	Inhibition of cell growth and proliferation, cell cycle arrest in the G1 phase in neuroblastoma cell lines in vitro	[56]
Dihydroartemisinin	SH-SY5Y	Induced apoptosis by decreasing the expression of cyclin D1 protein and increasing the expression of caspase-3 protein	[57]
Dihydroartemisinin	Lung (A549)	Induced apoptosis by increasing the ratio of Bax/Bcl-2 and active caspase-3 and cytochrome-c	[58]
		Inhibition of angiogenesis	[59]
Dihydroartemisinin	Human ovarian cancer (SKOV3, SKOV3-IP, HO8910, and HO8910-PM) and human ovarian surface epithelial cells))	Inhibited proliferation, migration, and invasion of ovarian cancer cells, and induced apoptosis in vitro	[60]
Artesunate		Induction of reactive oxygen species (ROS) cell cycle arrest in the G2/M phase	[61]
Dihydroartemisinin	Cervix carcinoma (HeLa)	Promoted autophagic cell death in vitro	[62]
Artemisinin		Artemisinin inhibited the G2/M phase	[63]
Dihydroartemisinin	Prostate (Human PCa cell lines C4, C4-2, and C4-2B)	Inhibits Axl expression in PCa via regulation of microRNAs and proteins of the polycomb repressive complex 2	[64]
Artesunate	DU145 and LNCaP	Inhibited the viability and mobility of the cell lines triggered by UCA1 down-regulation	[43]
Artesunate	Melanoma [Primary (92.1, Mel270) and metastatic (Omm1 and Omm2.3)]	Suppression of the phosphorylation of GSK3β at S9, and lowered protein level of β-catenin and its downstream targets (c-Myc, cyclin D1). Inhibition of cell viability and colony formation ability. Induced apoptosis with reduced migration and invasion of uveal melanoma cells. Induced upregulation of oxidative and genotoxic stress response genes	[65]
Dihydroartemisinin	A375, G361, LOX	Induced apoptosis with upregulation of cellular oxidative stress, phosphatidylserine externalization, and activational cleavage of procaspase 3.	[66]
Dihydroartemisinin	Hepatoma (HepG2 cell)	Induced apoptosis in HepG2 cell lines and increased the intracellular production of ROS	[67]
Artesunate	SMMC-7721	Induction of apoptosis and cell cycle arrest	[68]

**Table 2:** A summary of nanoparticles formulations of artemisinin and its derivatives designed for the treatment of cancer, malaria, and leishmaniasis.

Type of Nanoparticle	Carrier	Artemisinin Derivative	Application	Therapeutic Outcome	Ref.
Polymeric nanoparticles	Chitosan	Artemisinin	Anticancer	High drug loading capacity. Enhanced accumulation of the nanoparticles in the 4T1 breast tumor tissues of BALB/c mice model in vivo.	[122]
Polymeric nanoparticles	*N*,*N*′-bis(dodecyl)-l-glutamic diamide	Artesunate	Anticancer	The formulation mediated ROS generation and targeted the mitochondria, a target for inducing cancer cell death.	[123]
Polymeric nanoparticles	Bovine serum albumin	Artesunate	Anticancer	High cytotoxic effect and significant apoptotic effect.	[124]
Polymeric nanoparticles	Polyethylene glycol	Dihydroartemisinin		A significant growth inhibition effect with prolonged circulation time.	[126]
Polymeric nanoparticles	PLGA	Artesunate	Anticancer	Higher cytotoxicity against cancer cell lines in vitro.	[127]
Polymeric nanoparticles	PLGA	Dihydroartemisinin	anticancer	Sustained drug release kinetics and enhanced anticancer activity in vitro and in vivo.	[128]
Polymeric nanoparticles	PLGA	dihydroartemisinin	anticancer	High cell accumulation with enhanced cytotoxicity.	[129]
Lipid nanoparticles	cholesteryl oleatea and triolein	Dihydroartemisinin	Anticancer	A synergistic anticancer activity and high cell accumulation.	[130]
Lipid nanoparticles	egg phosphatidylcholine	Artemisinin	Anticancer	Effective against human breast cancer and non-toxic on non-tumorigenic cells.	[131]
Lipid nanoparticles	l-α-Phosphatidylcholine	Artemisinin	Anticancer	A down regulated of the anti-apoptotic protein, survivin, and cyclin D1 was observed in the breast cancer cell lines at low concentration of the formulation. A down regulated oncogenic protein HER2 and HER3 was observed in a HER2+ cell line with a reduction in the wild type epidermal growth factor receptor (EGFR or HER1) in a triple negative breast cancer cell line.	[132]
Lipid nanoparticles	Mannose-vitamin E derivative conjugate and a dequalinium-lipid derivative conjugate.	Artemether	Anticancer	Prolonged circulation time with a significant inhibitory effect and apoptosis-inducing effect against the brain cancer cells.	[133]
Lipid nanoparticles	Cholesterol	Artemisinin	Anticancer	Significant anticancer activity.	[134]
Lipid nanoparticles	Cholesterol	Artemisinin	Anticancer	The cell uptake and cytotoxicity studies of the formulation in HCT-8 cell line confirmed an enhanced uptake of the formulation due to the presence of iron ions.	[135]
Lipid nanoparticles	Soybean phosphatidylcholine, cholesterol,	Dihydroartemisinin	Anticancer	Downregulation of Bcl-xl, increased cancer cell apoptosis, and the induction of autophagy.	[136]
Lipid nanoparticles	Cholesterol, PEG	Dihydroartemisinin	Anticancer	High cellular uptake and the absence of toxicity	[137]
Lipid nanoparticles	Cholesterol	Artemether	Anticancer	High intracellular uptake and high generation of ROS in HepG2 cells.	[138]
Lipid nanoparticles	Cholesterol	Artemether	Anticancer	The in vitro drug release of artemether from the formulation was sustained. The growth inhibition rate was 1.54 times higher than the free drug solution.	[139]
Lipid nanoparticles	Lecithin, soy beans oil, poloxamer	Dihydroartemisinin	Anticancer	High tumor growth inhibition of 51.8% and extended half-life of the drugs.	[140]
Metal-based nanoparticle	Iron oxide	Dihydroartemisinin	Anticancer	Non-toxic and high anticancer efficacy in vitro.	[141]
Metal-based nanoparticle	CuS nanoparticles	Artesunate	Anticancer	A significant inhibition rate of 74.8% with a good tumor targeting ability and retention effect.	[142]
Metal-based nanoparticles	Iron oxide/silver	Artemisinin	Anticancer	Synergistic anticancer activity and good cellular uptake.	[145]
Metal-based nanoparticles	Iron oxide	Artesunate	Anticancer	Reduced cell viability.	[146]
Metal-based nanoparticles	Iron oxide	Dihydroartemisinin	Anticancer	Increased the amount of reactive oxygen species and significant killing effect on breast cancer cells, MCF-7 cells.	[147]
Polymeric nanoparticles	Graphene oxide	Dihydroartemisinin	Anticancer	A synergistic cytotoxicity activity with complete tumor cure.	[148]
Carbon-based nanoparticles	Carbon nanotubes	Artemisinin	Anticancer	A synergistic antitumor effect when compared to the free drug in vitro in MCF-7 cells and in vivo in tumor-bearing murine model. Increased intracellular drug uptake was significant with high inhibition effect.	[149]
Carbon-based nanoparticles	Fullerene	Artesunate	Anticancer	High drug loading efficacy of 162.4% and antitumor efficacy. The tumor inhibition rate.	[150]
Polymeric nanoparticles	PLGA	Artemisinin	Antimalarial	High drug encapsulation efficiency and controlled drug release mechanism.	[157]
Polymeric nanoparticles	PLGA	Artesunate	Antimalarial	Improved antimalarial activity in vivo with sustained and controlled drug release.	[158]
Polymeric nanoparticle	Cyclodextrin	Artemisinin	Antimalarial	Controlled drug release and parasite growth inhibition.	[159]
Polymeric nanoparticles	γ-cyclodextrin	Artemisinin	Antimalarial	Significant improved pharmacokinetic parameters.	[160]
Polymeric nanoparticles	Chitosan/lecithin	Artesunate and artemisinin	Antimalarial	Less mean percent parasitemia in vivo.	[161]
Lipid nanoparticles	Glyceryl monostearate	Artesunate	Antimalarial	Sustained drug release and enhanced drug intestinal permeability.	[162]
Lipid nanoparticles	steric acid	Dihydroartemisinin	Antimalarial	Good parasite chemosuppression in vivo.	[163]
Lipid nanoparticles	soybean oil (liquid lipid) and glyceryl trimyristate (as solid lipid)	Artemether	Antimalarial	Reduced hemolytic toxicity and good antiplasmodial efficacy in vivo.	[164]
Lipid nanoparticles	Human serum albumin	Artemether	Antimalarial	The nanoparticles displayed significantly enhanced solubility when compared to the free drug.	[165]
Lipid nanoparticles	Phospholipon^®^, theobroma oil and beeswax	Artemether	Antimalarial	High clearance of parasitemia with minimal side effects.	[166]
Lipid nanoparticles	PEG	Artemisinin	Antimalarial	Extended blood-circulation time and improved half-life of artemisinin by more than 5-fold.	[167]
Lipid nanoparticles	Glycerophosphorylcholine	Artesunate	Antimalarial	Longer retention half-life in the bloodstream. Enhanced parasites killing in *P. berghei*-infected mice in vivo with delayed recrudescence and improved survival when compared to free drug.	[168]
Lipid nanoparticles	Glucocorticoid prodrug	Artemisone	Antimalarial	Administration of artemisone after treatment with the liposome formulation resulted in a complete cure. The combination resulted in a reduced level of cerebral inflammation, hemorrhage and edema.	[169]
Lipid nanoparticles	Phosphatidylcholine	α/β Arteether	Antimalarial	High cure rate with the absence of recrudescence.	[170]
Lipid nanoparticles	-	Artemisinin	Antimalarial	The dissolution of drug-loaded nanoparticles was enhanced when compared to the free artesunate	[171]
Lipid nanoparticles	soybean oil, sodium oleate, glycerol, and egg lecithin, poloxamer	Artemether	Antimalarial	Decrease in the parasitemia levels after 3 days, and with parasitemia inhibition rate of 90%.	[172]
Lipid nanoparticles	Human serum albumin	Artesunate	Antimalarial	A 96% parasitemia inhibition at 10 mg/kg/day. Prolonged mean survival time with no recrudescence.	[173]
Lipid nanoparticles	soybean oil, oleic acid, egg lecithin	Artemether	Antimalarial	High Cmax of artemether and lumfantrine were 452.86 and 2844.15 ng/mL, respectively, in the lipid emulsion group when compared to the drug solution group which were 37.92 and 918.94 ng/mL.	[174]
Lipid nanoparticles	l-α-Phosphatidylcholine, Labrasol	β-arteether	Antimalarial	Increased survival rate and a significant delayed recrudescence.	[175]
Lipid nanoparticles	Tween 80, PEG 400	Arteether	Antimalarial	Improved drug bioavailability.	[176]
Lipid nanoparticles	groundnut oil, Tween 80	β-arteether	Antimalarial	A 100% cure for more than 45 days.	[177]
Lipid nanoparticles	Soybean oil	Artemether	Antimalarial	Excellent antimalarial activity with regards to parasitemia progression and survivability period.	[178]
Metal-based nanoparticles	Iron oxide	Artesunate	Antimalarial	Good intracellular drug uptake with the improved antimalarial activity.	[179]
Polymeric nanoparticles	PLGA	Artemisinin	Antileishmanial	Sustained in vitro drug release and ex vivo antileishmanial activity	[182]
Polymeric nanoparticles	PLGA	Artemisinin	Antileishmanial	Significant parasite burden reduction	[183]
Lipid nanoparticles		Artemisinin	Antileishmanial	Significantly reduced intracellular infection of Leishmania donovani amastigotes.	[184]
